# Combined Transcriptome and Metabolome Analysis of Alfalfa Response to Thrips Infection

**DOI:** 10.3390/genes12121967

**Published:** 2021-12-10

**Authors:** Zhiqiang Zhang, Qi Chen, Yao Tan, Shuang Shuang, Rui Dai, Xiaohong Jiang, Buhe Temuer

**Affiliations:** 1 Key Laboratory of Grassland Resources of the Ministry of Education, Technology Engineering Center of Drought and Cold-Resistant Grass Breeding in North of the National Forestry and Grassland Administration, College of Grassland, Resources and Environment, Inner Mongolia Agricultural University, Hohhot 010011, China; zhangzq1989@imau.edu.cn (Z.Z.); ndcq@emails.imau.edu.cn (Q.C.); bss@emails.imau.edu.cn (S.S.); jxh19971002@sina.com (X.J.); 2 Key Laboratory of Grassland Resources of the Ministry of Education, College of Grassland, Resources and Environment, Inner Mongolia Agricultural University, Hohhot 010011, China; 18747659997@163.com; 3 College of Horticulture and Plant Protection, Inner Mongolia Agricultural University, Hohhot 010011, China; 850310.tanhuaf4@163.com

**Keywords:** transcriptome, metabolome, *Medicago sativa*, thripidae, induced defense

## Abstract

Thrips (Thysanoptera: Thripidae) is a major insect pest for alfalfa which can result in decreased plant nutrients, low yields, and even plant death. To identify the differentially expressed genes and metabolites in response to thrips in alfalfa, a combination of metabolomics and transcriptomics was employed using alfalfa (Caoyuan No. 2) with and without thrips infestation. The results showed that the flavonoid biosynthesis and isoflavonoid biosynthesis pathways were the most significantly enriched pathways in response to thrips infection, as shown by the combined transcriptome and metabolome analysis. The transcriptome results showed that SA and JA signal transduction and PAPM-triggered immunity and the MAPK signaling pathway–plant pathways played a crucial role in thrips-induced plant resistance in alfalfa. In addition, we found that thrips infestation could also induce numerous changes in plant primary metabolism, such as carbohydrate and amino acid metabolism as compared to the control. Overall, our results described here should improve fundamental knowledge of molecular responses to herbivore-inducible plant defenses and contribute to the design of strategies against thrips in alfalfa.

## 1. Introduction

Alfalfa (*Medicago sativa* L.), a legume forage with high quality and yield, has become an important material basis for the development of grass husbandry all over the world. Herbivorous thrips (Thysanoptera) are important pests affecting alfalfa in China and most other regions where alfalfa is grown [1,2]. Thrips feed on leaves, stems, fruits, and/or pollen of green plants, and they are vectors of destructive viruses, resulting in decreased plant nutrients and growth, low yields, and even plant death [3]. It has been reported that thrips cause about 10%–30% grass yield loss every year [4]. However, thrips are highly polyphagous and hard to control due to their complex lifestyle [3]. Integrated pest management guidelines for crops emphasize use of a range of tactics to reduce pest abundance, rather than reliance on insecticides, which would induce ecosystem damage and food safety issues [5].

Plants have developed highly effective and dynamic defensive strategies against insect pests, including various morphological and biochemical defenses that restrict insect pests, including constitutive defense and induced defense [6,7]. Compared with constitutive defense, induced defense is more often initiated upon herbivore attack due to the trade-off between plant growth and defense [8]. Herbivore-inducible plant defenses are initiated after perception of the herbivore through damage-associated molecular patterns or herbivore-associated molecular patterns. Many authors have reported that several morphological traits were associated with host plant resistance to thrips, such as leaf hair density, leaf hairiness, leaf hardness, leaf wax, glandular hairs, and trichomes [9,10,11]. However, biochemical-based defense is considered more effective, as it directly affects insect growth and development [12]. It has been reported that many metabolites and plant hormones are associated with thrips resistance, such as protease inhibitors, phenols, tannins, salicylic acid (SA), and jasmonic acid (JA) [13,14,15,16]. Most of the metabolites known to be involved in thrips constitutive defense are inherently present in the plant [17,18,19,20,21], but some accumulate in response to thrips infestation. For instance, phenolic compounds, such as tannin, have been found to accumulate in response to thrips infection [7,20].

Compared to leaf-chewing or phloem-feeding herbivores, far less is known about the induced plant responses to cell-content-feeding insects such as thrips [8]. Even though there is literature related to constitutive defense mechanisms against thrips [17,18,19,21], functional analyses of defensive compounds that are induced upon thrips attack are less [3,20]. Next generation sequencing, and more specifically RNA-Sequencing (RNA-Seq), has become a popular and comprehensively informative approach to predict and validate novel key regulators and their direct and indirect targets in plant signaling networks to pests. Recently, a comparative transcriptomes analysis was employed to assemble the expressed genes of alfalfa, which mainly focused on the induced defense genes related to both resistant and susceptible alfalfa lines after thrips infestation, but the analysis paid little attention to the genes specially related to thrips resistance in the resistant cultivar [22]. In addition, metabolomics can aid the discovery of plant metabolites related to thrips induced resistance. Combined transcriptome and metabolome can allow quantitative mapping of transcripts directly to metabolic pathways involved in thrips induced resistance.

It is important to understand the metabolic changes, transcriptional regulation, and physiological responses of bioactive and signaling compounds during infection of alfalfa with thrips. Thus, to identify the differentially expressed genes and metabolites in response to thrips in alfalfa, a combination of metabolomics and transcriptomics was employed using alfalfa (Caoyuan No. 2) with and without thrips infestation. The results showed that the flavonoid biosynthesis and isoflavonoid biosynthesis pathways were the most significantly enriched pathways in response to thrips infection, as shown by the combined transcriptome and metabolome analysis. The transcriptome results showed that plant hormones signal transduction (SA and JA) PAPM-triggered immunity and that MAPK signaling pathway–plant pathways played a crucial role in thrips-induced plant resistance in alfalfa. In addition, we found that thrips infestation could also induce numerous changes in plant primary metabolism, such as carbohydrate and amino acid metabolism as compared to the control. Overall, our results described here should improve fundamental knowledge of molecular responses to herbivore-inducible plant defenses and contribute to the design of strategies against thrips in alfalfa.

## 2. Materials and Methods

### 2.1. Plant Growth and Thrips Infection

Seeds of alfalfa (Caoyuan No. 2) were cultivated in pots (H21 cm × D14 cm, one plant per pot) containing field collected soil in a greenhouse with a relative humidity of 60 ± 5% and 70 ± 5% at 30 ± 5 °C and 20 ± 5 °C during day and night, respectively. Plants were watered every other day. Both cultivars were bred at Inner Mongolia Agricultural University, China. Alfalfa plants were treated as described by Tu et al. [22] with some modifications. When the seedlings reached budding stage (about 60 days), they were randomly and equally divided into two groups: (1) 30 alfalfa thrips per plant were placed onto the leaves and covered by a cage with a 90-mesh nylon cloth as the S_T treatment group; and (2) plants were not treated with thrips and were maintained under the same conditions, as the S_CK treatment group. Three plants were grown in each pot, and 4 pots were counted as one biological replicate. After three weeks, the top 3–4 leaves were cleaned of any thrips and harvested from each treatment. All samples were immediately frozen in liquid nitrogen and stored at −80 °C.

### 2.2. RNA Extraction, cDNA Library Construction and RNA-Sequencing

Three biological replicates were used for all RNA-Seq experiments from thrips and no-thrips treatments. RNA extraction, cDNA library construction and RNA-sequencing were carried out as described by [13,23]. Briefly, total RNA was extracted from the leaves using Trizol reagent (Invitrogen, Carlsbad, CA, USA) along with DNase treatment, according to the manufacturer’s instructions (QIAGEN, Hilden, Germany). The quality and quantity of total RNA were assessed using NanoDrop 2000 analysis and gel electrophoresis. As described by [24], the cDNA libraries were prepared using a TruseqTM RNA sample prep Kit (Illumina, San Diego, CA, USA), and RNA-sequencing was performed on an Illumina Hiseq 4000 (Version 2 × 150 bp) at Shanghai Majorbio Bio-pharm Biotechnology Co., Ltd. (Shanghai, China). The raw sequence reads were deposited in the NCBI Sequence Read Archive (http://www.ncbi.nlm.nih.gov/Traces/sra, accession number PRJNA622603) (accessed on 3 April 2020).

### 2.3. De Novo Assembly, Annotation and Classification

The clean data were obtained by removing the adapter and primer sequences using SeqPrep software, and fragments of less than 20 bp in length were excluded from further analyses using software Trinity (https://github.com/trinityrnaseq/trinityrnaseq) (accessed on 25 September 2021) in the absence of a reference genome [25]. The sequence assembly quality was evaluated using the number of sequences and bases, GC percentage, distribution of unigene lengths, average coverage, and N50 statistics [26].

The assembled transcriptome sequences were searched against six databases (NR, Swiss-Prot, Pfam, COG, GO, and KEGG databases) to obtain annotation information in each database. Specifically, to obtain the similarity to other species and the functional information of homologous sequences, the sequences were searched against the NCBI non-redundant database (NR, ftp://ftp.ncbi.nlm.nih.gov/blast/db/, accessed on 25 September 2021), Swiss-Prot database (http://web.expasy.org/docs/swiss-prot_guideline.html, accessed on 25 September 2021), and Pfam (http://pfam.xfam.org/, accessed on 25 September 2021). The gene function terms were obtained through the Gene Ontology database (GO, http://www.geneontology.org, accessed on 25 September 2021). Functional classification was performed using the Clusters of Orthologous Groups of proteins database (COG, http://www.ncbi.nlm.nih.gov/COG/, accessed on 25 September 2021), and pathway annotation was performed using the Kyoto Encyclopedia of Genes and Genomes (KEGG, http://www.genome.jp/kegg/, accessed on 25 September 2021). A *p*-value ≤ 0.05 was regarded as the threshold for significance [26]. In addition, quantitative analysis of gene and transcript expression levels was obtained through RSEM (http://deweylab.github.io/RSEM/, accessed on 25 September 2021), and principal component analysis (PCA) was performed to obtain the relationships among and variability between samples.

### 2.4. Differentially Expressed Genes Analysis and Enrichment Analysis

Differential expression gene analysis of the samples was performed using DEseq 2 software. A *p*-adjusted value <0.05 and |log2FC| ≥ 1 was set as the threshold. To identify shared and unique genes/transcripts across gene sets, Venn analysis was performed. A gene set enrichment analysis of the differentially expressed genes (DEGs) was then performed for the KEGG annotations to determine over-represented functional pathways (with a *p*-value < 0.05) at each comparison level for different genotypes and treatments.

### 2.5. Metabolome Analysis

The sample processing, extraction, and metabolites detection for metabolome analysis were performed on Wuhan MetWare Biotechnology Co., Ltd. (Wuhan, China) following their standard procedures [27].

### 2.6. Metabolomics Data Analysis

Data matrices with the intensity of metabolite features with and without thrips infection were submitted and processed in Analyst 1.6.3 software (AB SCIEX, Concord, ON, Canada). The missing values were considered to be below the detection limit and imputed with a minimum recorded value [28]. The ion intensities were normalized by log transformation, metabolite abundance was calculated by using Dunnett’s test, and multiple testing was controlled by fold change ≥2 and fold change ≤0.5. We used the quadrature signal correction partial least squares-discriminant analysis (OPLS-DA) and the variable importance in projection (VIP) to obtain the maximum differences between control and thrips infection. Metabolites with VIP > 1.0 were considered as differential metabolites for group discrimination. The KEGG database [28] was used to annotate and display the differential metabolites. Other analyses included Principal Component Analysis (PCA) and pathway enrichment, which were completed using R as reported (www.rproject.org, accessed on 20 June 2020) [27].

### 2.7. Combined Transcriptome and Metabolome Analyses

We performed co-joint analyses on the differentially expressed genes and differentially accumulated metabolites to determine the degree of enrichment of pathways. Gene-metabolite networks with a Pearson correlation coefficient (PCC) > 0.8 were used to construct the transcript-metabolite network [29].

## 3. Results

### 3.1. Summary of Transcriptome and Metabolome Analysis

**Figure 1:**
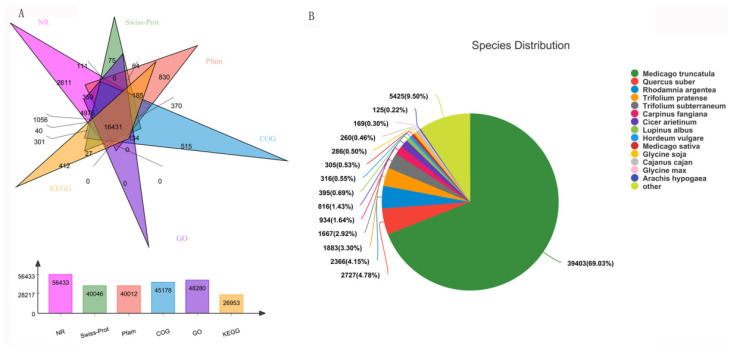
Function annotations of transcriptome sequencing. (**A**) Functional annotation numbers of unigenes in the NR, Swiss-prot, Pfam, COG, GO, and KEGG databases. (**B**) Annotated species distribution in the NR database.

Transcription and widely targeted metabolites profiles of Caoyuan No. 2 without or with thrips treatment (S_CK vs. S_T) were explored. Three independent biological replicates were used for each treatment, resulting in six samples. A total of 86.34 Gb Clean Data was obtained and the clean data of each sample reached more than 12.57 Gb, and the Q30 base percentage was more than 91.67%. A transcriptome database containing 99,111 unigene of average length 822.74 bp was obtained using Trinity software, with an N50 length of 1267 bp and an E90N50 length of 2172 bp. All unigenes and transcripts obtained by transcriptome assembly were aligned with six major databases (Nr, Swiss-prot, Pfam, COG, GO, and KEGG databases). A total 62,266 homologs of the 99,111 assembled unigenes were found to have homologs in the databases NR (56,433), Swiss-prot (40,046), Pfam (40,012), COG (45,178), GO (48,280), and KEGG (26,953) (Figure 1A). For the species distribution of the top BLAST hits in the NR database, 39,403 (69.03%) annotated unigenes matched the sequence of *Medicago truncatula* (Figure 1B). In addition, 772 metabolites were detected, which could be grouped into 23 major classes (Table S1).

**Figure 2:**
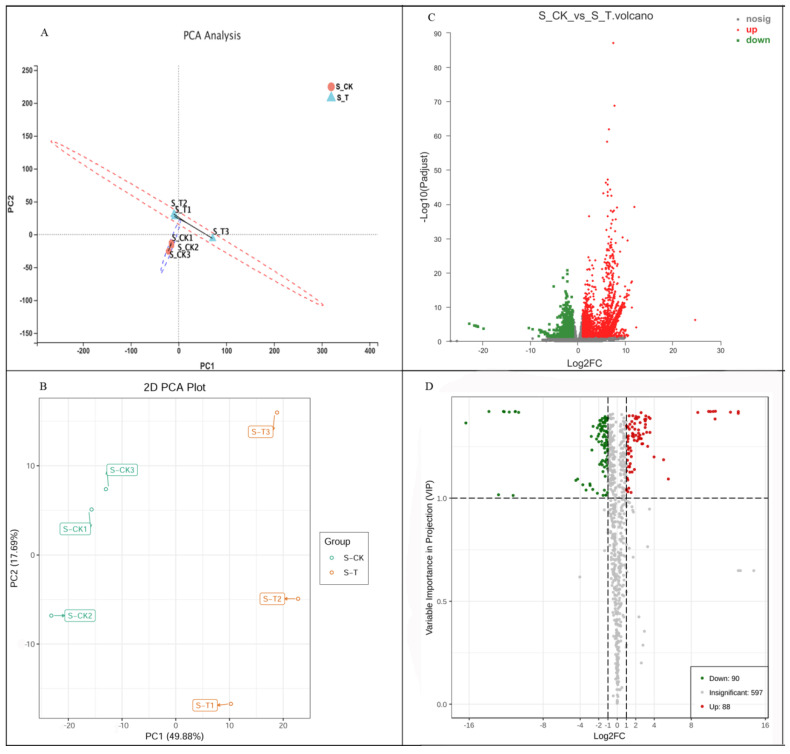
Differentially expressed genes and differentially accumulated metabolites between S_CK and S_T. (**A**) Principal component analysis (PCA) of the variance-stabilized estimated raw counts of differentially expressed genes. (**B**) Principal component analysis (PCA) of the variance-stabilized estimated raw counts of differentially accumulated metabolites. (**C**) Expression profiling changes of genes in thrips injection tissues. (**D**) Volcano Plot of differentially expressed genes between S_CK and S_T. Each point in the volcano map represents a metabolite, the X axis represents the logarithm of the quantitative difference of a certain metabolite in the two samples; the Y axis represents the VIP value. Samples are categorized by cultivars and thrips infestation as different marker colors and shapes. The green dots represent downregulated differentially expressed genes or differentially accumulated metabolites, the red dots represent upregulated differentially accumulated metabolites or genes, and the gray represents detected but not significantly differentially expressed genes or differentially accumulated metabolites. S_CK: Caoyuan No. 2 without thrips infection; S _T: Caoyuan No. 2 with thrips infection.

Principal component analysis (PCA) of the differentially expressed genes and differentially accumulated metabolites showed that the S_CK treatment group showed obvious differences with the S_T treatment group, which explained 44.32% and 49.88% of the total variation (Figure 2A,B). These results indicated that Caoyuan No. 2 is susceptible to thrips.

### 3.2. Differentially Expressed Genes and Differentially Accumulated Metabolites Analysis Related to Thrips Infection

The differentially expressed genes among the two groups were analyzed in the RNA-seq datasets. After treatment with thrips, a total of 4187 DEGs were detected, of which, respectively, 3379 and 808 upregulated and downregulated differentially expressed genes were observed (S_CK vs. S_T, *p*-adjust value < 0.05 and |log2FC| ≥ 1, Figure 2C). For the evaluation of differentially accumulated metabolites between S_CK and S_T, the OPLS-DA model was applied. The established OPLS-DA model showed good fitness (Figure S1). After treatment with thrips, a total of 88 upregulated and 90 downregulated metabolites were detected between the treatments (S_CK vs. S_T, VIP > 1 and |log2FC| ≥ 1, Figure 2D).

**Figure 3:**
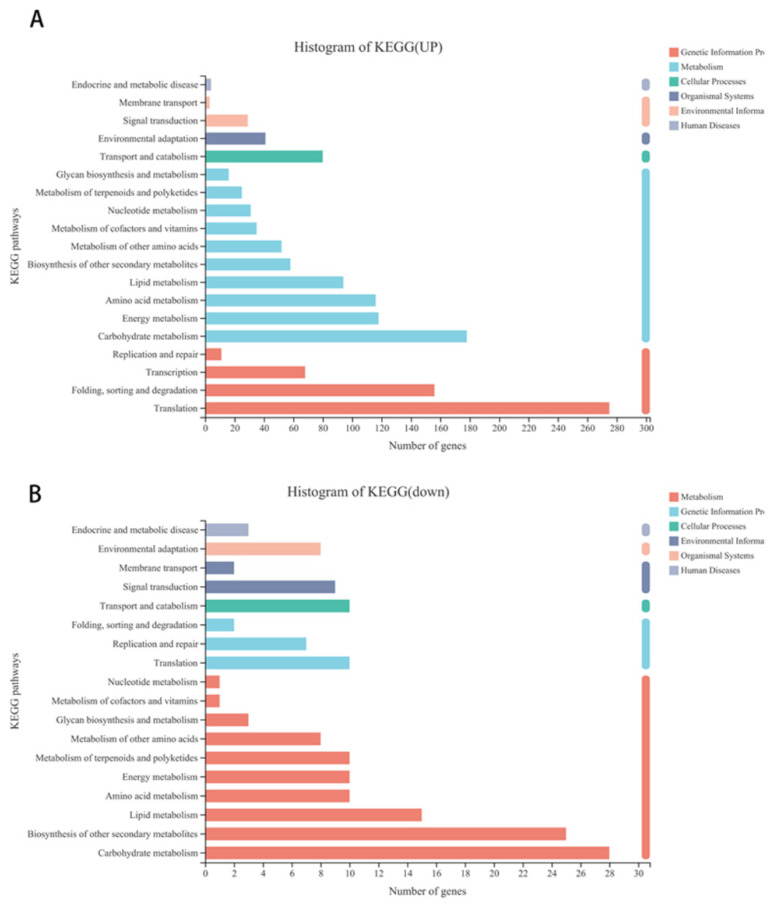
KEGG pathway classification of differentially expressed genes in alfalfa after thrips infection. (**A**) KEGG pathway classification of upregulated differentially expressed genes in alfalfa after thrips infection. (**B**) KEGG pathway classification of downregulated differentially expressed genes in alfalfa after thrips infection.

Further analysis showed that 1681 upregulated and 199 downregulated genes were annotated to 116 and 74 different KEGG pathways, respectively (Table S2). Further analysis showed that 499 of the upregulated and 19 of the downregulated differentially expressed genes were annotated to pathways including genetic information process (such as folding, sorting, and degradation transcription, translation), 723 of the upregulated and 111 of the downregulated differentially expressed genes were related to metabolism (such as carbohydrate metabolism, lipid metabolism, amino acid metabolism, biosynthesis of other secondary metabolites, etc.), 80 of the upregulated and 10 of the downregulated differentially expressed genes were involved in cellular process, and 41 and 29 upregulated as well as 9 and 10 of the downregulated differentially expressed genes were annotated to environmental adaptation and signal transduction, respectively (Figure 3A,B, Table S2).

Furthermore, we found that all the key genes (*NPR1, TGA*, and *PR-1*) related to salicylic acid (SA) transduction and *JAZ* gene related to jasmonic acid (JA) signal were significantly induced in S_CK after thrips attack (Figure S2). In addition, the results showed that some key genes involved in plant–pathogen interaction (such as *elfl8*, *CDPK, Rboh, CaMCML, MPK4, WRKY22, WRKY25, WRKY29, WRKY33, Pit6, HSP90, SGT1, EDS1, NHO1, PR1*, and *KCS*) and MAPK signaling pathway–plant (such as *MPK4, WRKY22, WRKY29, WRKY33, ACS6, NDPK2, CaM4, Rboh*, and *PR1*) were induced or suppressed by thrips infection (Figures S3 and S4).

**Figure 4:**
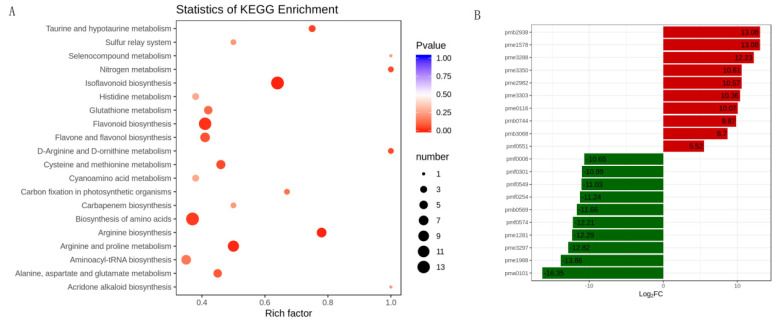
The differentially accumulated metabolites analysis in alfalfa after thrips infection. (**A**) Scatter plot of KEGG pathways in alfalfa after thrips infection to which the differentially accumulated metabolites were enriched. The degree of enrichment is shown by Rich factor, *p*-value, and the number of metabolites enriched in each pathway; (**B**) Top 10 up-accumulated (shown in red bars) and top 10 down-accumulated metabolites (shown in green bars) in alfalfa after thrips infection.

For differentially accumulated metabolites, we found that the differentially accumulated metabolites were most significantly enriched to flavonoid biosynthesis, isoflavonoid biosynthesis, amino acids biosynthesis, and arginine and proline biosynthesis (Figure 4A). Furthermore, the results showed that the top 10 upregulated metabolites in alfalfa after thrips infection were 8-Hydroxy-2-deoxyguanosine (pmb3350), 4-*O*-Caffeoyl quinic acid (pme2938), genistein (pme1578), 3,7-Di-*O*-methylquercetin (pme3288), 8-Hydroxy-2-deoxyguanosine (pme3350), pinocembrin (pme2982), tricetin (pme3303), l-Carnosine (pme0116), tricin *O*-phenylformic acid (pmb0744), 1-*O*-p-Coumaroyl quinic acid (pmb3068), and tectochrysin (pmf0551). The top 10 downregulated metabolites between S_CK and S_T were N-Caffeoyl agmatine (pma0101), l-Alanine (pme1988), kaempferol 3-*O*-rhamnoside (pme3297), pyridoxal 5’-phosphate (pme1281), sucralose (pmf0574), syringetin (pmb0569), 5-*O*-hexoside, solanine (pmf0254), diosmin (pmf0549), engeletin (pmf0301), and narirutin (pmf0006) (Figure 4B).

### 3.3. Combined Transcriptome and Metabolome Analyses

**Figure 5:**
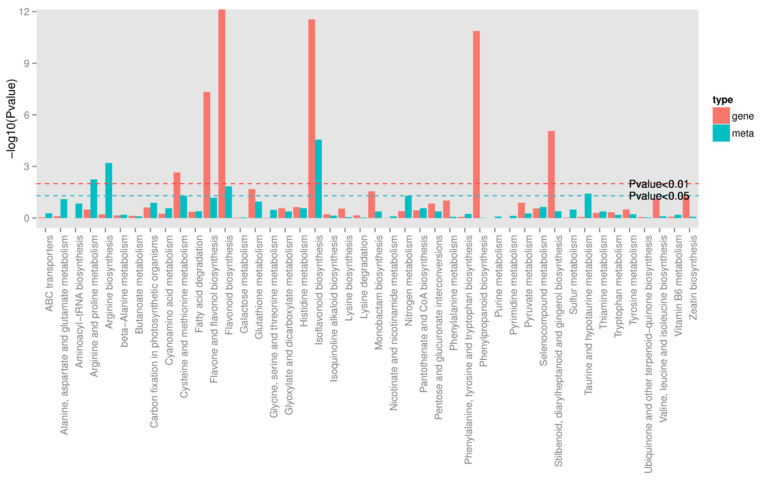
Joint analysis of the differentially expressed genes and differentially accumulated metabolites between S_CK and S_T. Green line represents the selected gene and metabolic pathway at *p*-value < 0.05, and red line represents the selected gene and metabolic pathway at *p*-value < 0.01.

**Figure 6:**
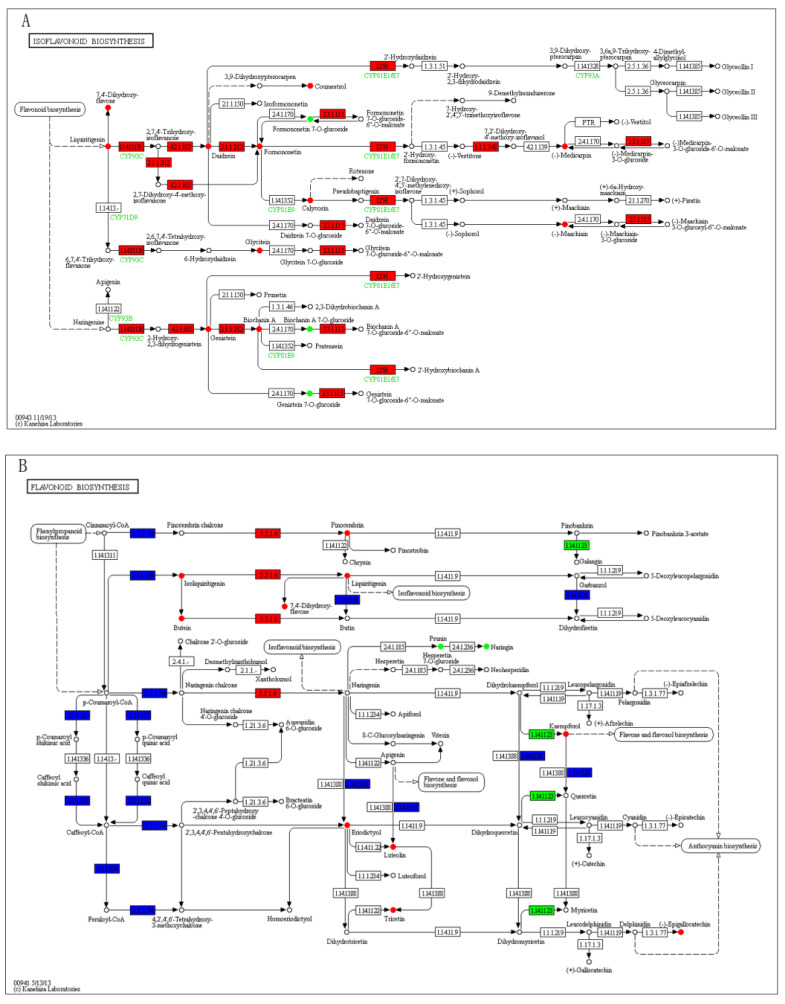
The differentially expressed genes and differentially accumulated metabolites simultaneously mapped to the KEGG pathway diagram. (**A**) The differentially expressed genes and differentially accumulated metabolites simultaneously mapped to the isoflavonoid biosynthesis (ko00943). (**B**) The differentially expressed genes and differentially accumulated metabolites simultaneously mapped to the flavonoid biosynthesis (ko00941). Green color represents a significant downregulation of the gene or metabolite, red color represents a significant upregulation of the gene or metabolite, and blue color represents a gene that is both upregulated and downregulated.

To quantitatively map the transcripts directly to metabolic pathways involved in thrips induced resistance, the co-jointKEGG pathway enrichment analysis of transcriptome and metabolome was performed. The results showed that the same pathways of DEGs and DAMs were enriched to flavonoid biosynthesis (*p*-value < 0.05) and isoflavonoid biosynthesis (*p*-value < 0.01) (Figure 5). In order to better understand the relationship between genes and metabolites, the differentially expressed genes and differentially accumulated metabolites were simultaneously mapped to the KEGG pathway diagram (Table S4). As shown in Figure 6A,B, nine key upregulated genes (*CYP93C, HI40MT, HIDH, I2′H, IF7MAT, 7-IOMT, VR, CYP81E9,* and *PTR*) and 14 metabolites (11 upregulated and 3 downregulated) were simultaneously mapped to the isoflavonoid biosynthesis (ko00943). In addition, 14 differentially accumulated metabolites and 2 key the differentially expressed genes were simultaneously mapped to the flavonoid biosynthesis (ko00941).

## 4. Discussion

Alfalfa is an extremely energy efficient crop and is playing an increasingly important role in low input sustainable agriculture. However, more than 100 insect species damage alfalfa in southeast Asia, northeast Africa, and the U.S. [30]. Thus, it is important to use insect-resistant cultivars to control insects that damage both the quantity and quality of the alfalfa. Plants have evolved effective defense mechanisms against insect infestation including morphological traits [31,32], mechanistic (trichomes, hairs) defenses and chemical defenses that involve genes and pathways related to diverse mechanisms [3,33]. Recently, high-quality genome of thrips [34] and various ‘omic’ technologies [8] have been reported, which may deepen our understanding of the interaction between thrips and plants. For breeding insect resistance and insect control in crops, it is necessary to have information on genetic variation in the host reaction to insect infestation [35]. In this study, we studied the differentially expressed genes and differentially accumulated metabolites based on combined transcriptome and metabolome profiling of S_CK and S_T of Caoyuan No. 2 to explore the mechanisms related to thrips-induced plant resistance.

### 4.1. Primary Metabolites Changed Related to Thrips Infestation

It has been reported that numerous changes in plant primary metabolism, such as carbohydrate and nitrogen metabolism, occur in response to insect attacks [36], and amino acid composition and level affect plant insect resistance [37]. Amino acids are a major source of nitrogen, their content in sap act as a limiting factor in determining the survival of insects [38]. Many plants’ defensive compounds are derived from amino acid precursors such as secondary metabolites and glucosinolates [39]. In this study, 23 amino acid and derivatives were significantly changed in alfalfa after thrips infection. Specifically, the upregulated amino acid and derivatives were mainly toxic amino acids or sulphur-amino acid such as l-Carnosine, Phenylacetyl-l-glutamine, l-Cysteine, dl-Homocysteine, *N*-Phenylacetylglycine, and l-Kynurenine, which might play a crucial role in thrips induced plant defense (Table S3). Consistent with this finding, numerous amino acid metabolism genes related to these amino and derivatives were induced by thrips infestation (Table S2). In addition, plant epicuticular lipid extracts and individual lipid components such as cutin and wax are important for plant insect resistance by affecting oviposition, movement, and feeding [40,41,42]. One interesting finding in our study was that all the different lipids between S_CK and S_T were downregulated (Table S3). This indicated that lipid levels were negatively correlated with the thrips induced plant defense. Increased photosynthesis and/or local carbohydrate catabolism can serve as energy sources for the production of plant defenses plant–herbivore interactions [43,44]. Consistent with this literature, our study found that many genes involved in energy metabolism (including oxidative phosphorylation and carbon fixation in photosynthetic organisms) and carbohydrate metabolism were induced by thrips infestation (Table S1). These results reveal that primary metabolite (i.e., carbohydrate metabolism, lipid metabolism, and amino acid metabolism) pathways play important roles in thrips induced defense in alfalfa.

### 4.2. Hormones Signaling Pathways Related to Thrips Infestation

Jasmonic acid (JA) and salicylic acid (SA) are the main signal-transduction pathways in plants [45], underlying induced defense against attackers such as herbivorous insects. Both signaling pathways usually act antagonistically, but also have been reported to act synergistically or additively [46,47]. In this study, key genes related to SA transduction including NPR1, TGA, and PR-1 were significantly upregulated (Figure S2). In accordance with the present results, previous studies have demonstrated both thrips resistant and susceptible alfalfa cultivars can regulate gene expression in the SA pathways to enhance plant defense capacity [22]. Thrips feeding activities have been shown to activate the expression of JA-responsive genes [33,48]. Our results showed that thrips feeding activated the *JAZ* gene (Figure S2), which is one of the key genes in SA biosynthesis. Except for direct damage caused by feeding, thrips also serve as vectors for plant diseases such as tospoviruses. Some findings indicate that viruses can interfere with plant defenses through the interaction of SA with JA signaling [49]. Our results indicated that the SA and JA signaling pathways play important roles in thrips induced plant defense.

### 4.3. Plant Immunity Signaling Pathways Related to Thrips Infestation

It is well known that plant–pathogen and plant–insects interactions share some responses. Pathogen-associated molecular patterns (PAMPs) and herbivore-associated molecular patterns (HAMPs) are recognized by receptors on the cells in different part of the plant which activate the defense signaling pathways, resulting in a plant’s ability to overcome pathogenic invasion and protect against insect predation and damage [47]. We found that the majority of DEGs related to fungal PAPM, bacterial EF-Tu, and bacterial secretion, and that the MAPK signaling pathway–plant were induced in Caoyuan No. 2 after thrips attack (Figures S3 and S4). The activity of these genes would result in plant programmed cell death, the maintenance of the homeostacis, or the accumulation of reactive oxygen species, hypersensitive response, cell wall reinforcement, and stomatal closure and defense-related gene induction. Thus, the thrips induced plant defense occurred. These results indicated that the differentially expressed genes related to MAPK signaling and plant–pathogen interaction may be important for plant-induced defense to thrips, which is consistent with our previous studies and other reports [50,51].

### 4.4. Plant Secondary Metabolites Pathway Related to Thrips Infestation

Plant secondary metabolites, such as alkaloids, glucosinolates, or phenolic compounds, serve as plant defenses against insects [52,53,54]. However, the content and distribution of individual secondary metabolites vary greatly among plant genotypes [55]. In our study, the results of both transcriptome and metabolome analyses showed that flavonoid biosynthesis and isoflavonoid biosynthesis pathways were induced by thrips injection (Figure 5 and Figure 6A,B). This finding is consistent with other studies which observed that both thrips resistant and susceptible alfalfa cultivars can regulate gene expression in the flavonoid biosynthesis pathways to induce defensive genes and protein expression [22]. Similar results have been revealed in chickpea and common bean infected with different pathogens [56,57]. Hence, the induction of flavonoid biosynthesis related genes and metabolites in S_T suggested their potential involvement in thrips induced plant defense in alfalfa.

## 5. Conclusions

Thrips feeding could induce several changes in alfalfa. The flavonoid biosynthesis and isoflavonoid biosynthesis pathways were the most significantly enriched pathway in response to thrips infection as shown by the combined transcriptome and metabolome analysis. Plant hormones signal transduction (SA and JA), PAPM-triggered immunity and MAPK signaling pathway–plant pathways played a crucial role in thrips-induced plant resistance in alfalfa. In addition, we found that thrips infestation could also induce numerous changes in plant primary metabolism, such as carbohydrate and amino acid metabolism as compared to the control. Overall, the results described here improve fundamental knowledge of molecular responses to herbivore-inducible plant defense and contribute to the design of strategies against thrips in alfalfa.

## Data Availability

Data is contained within the article and Supplementary Material.

## Supplementary Materials

The following are available online at https://www.mdpi.com/article/10.3390/genes12121967/s1, Figure S1: The quadrature signal correction partial least squares-discriminant analysis (OPLS-DA) between S_CK and S_T. Figure S2: Differentially expressed genes between S_CK and S_T related to SA and JA transduction pathway. Figure S3: Differentially expressed genes between S_CK and S_T related to plant–pathogen interaction pathway. Figure S4: Differentially expressed genes between S_CK and S_T related to MAPK signaling pathway–plant pathway. Table S1: All metabolites detected by metabolome. Table S2: Up and downregulated genes between S_CK and S_T annotated to different KEGG pathways. Table S3: Differentially accumulated metabolites between S_CK and S_T. Table S4: The differentially expressed genes and differentially accumulated metabolites and simultaneously mapped to the KEGG pathway diagram (ko00941 and ko00943) in details.
