# Supplementary figures and images for: Combined Transcriptome and Metabolome Analysis of Alfalfa Response to Thrips Infection
Source: Genes (Basel). 2021 Dec 10;12(12):1967. doi: 10.3390/genes12121967 (PMC8701657; doi:10.3390/genes12121967)

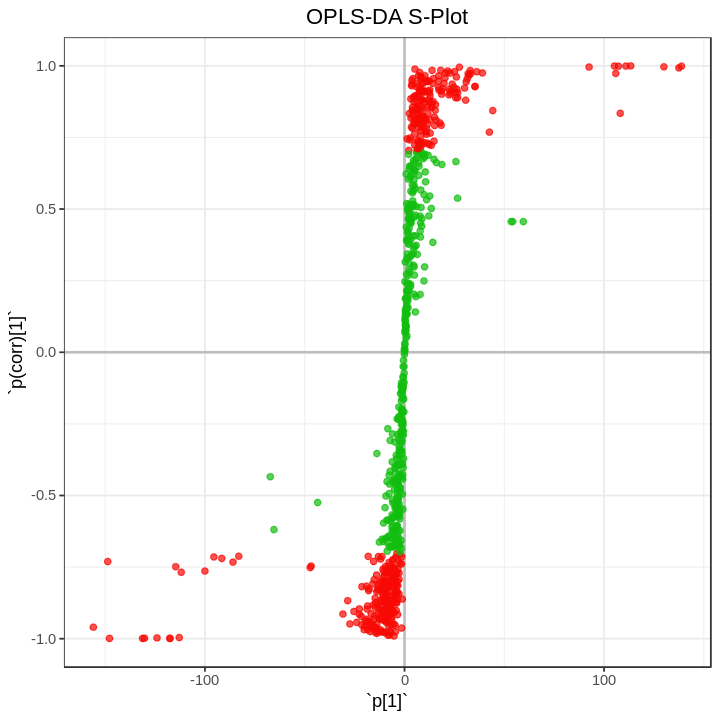

Supplement: Supplementary file 1 [file genes-12-01967-s001.zip › Figure S1.png]

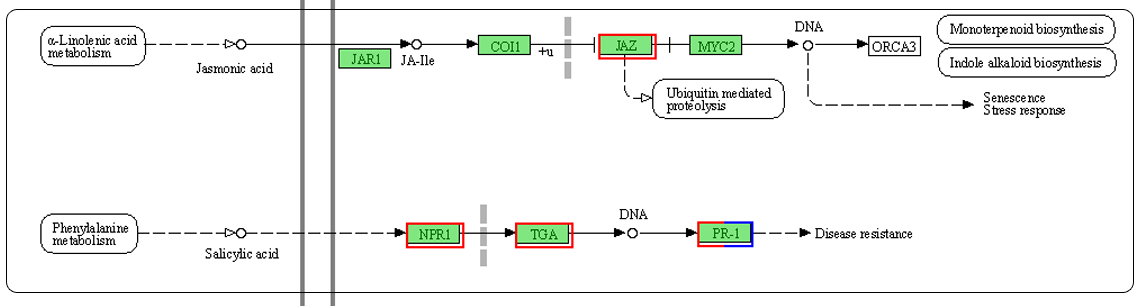

Supplement: Supplementary file 1 [file genes-12-01967-s001.zip › Figure S2.tif]

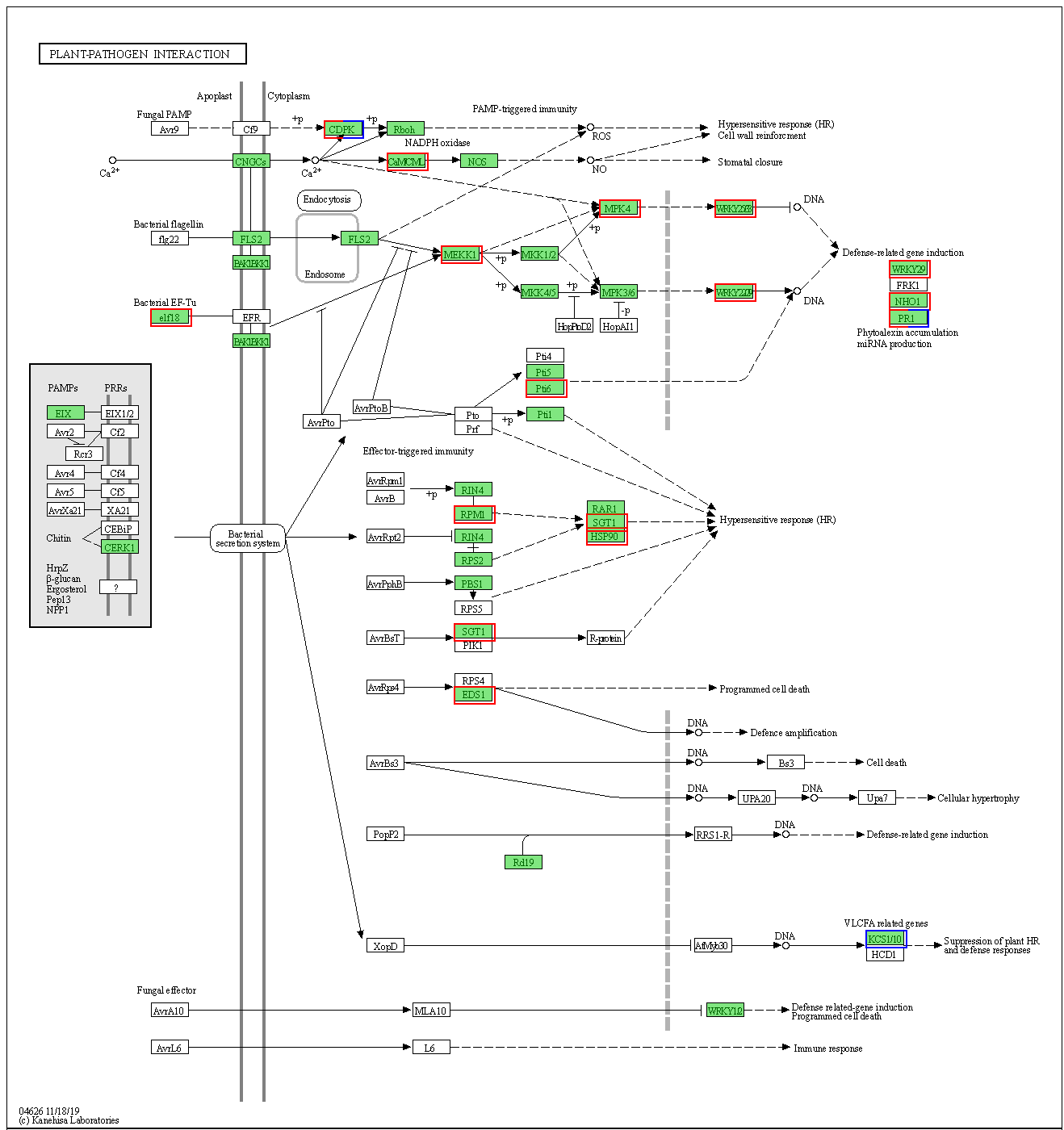

Supplement: Supplementary file 1 [file genes-12-01967-s001.zip › Figure S3.png]

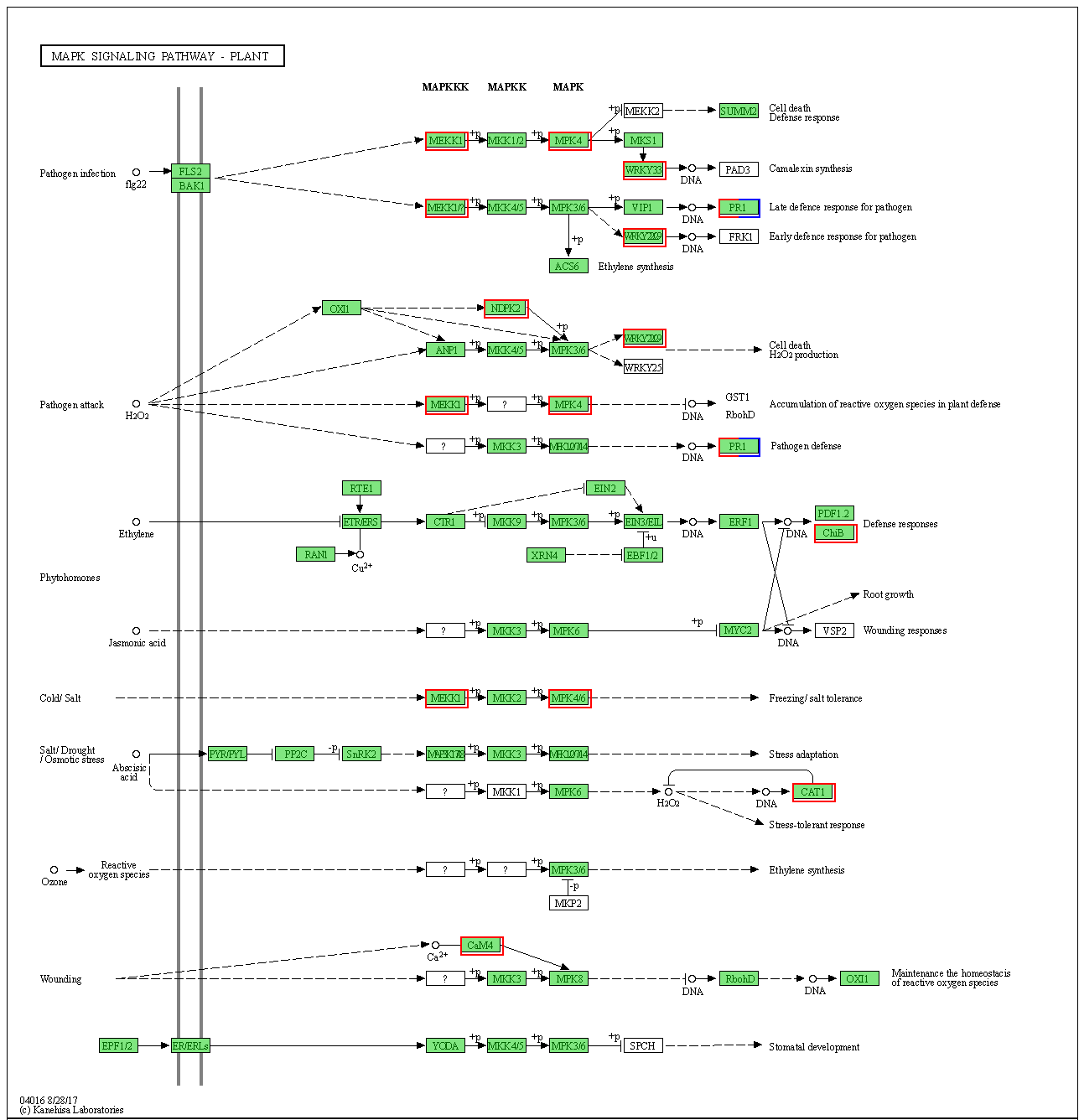

Supplement: Supplementary file 1 [file genes-12-01967-s001.zip › Figure S4.png]
